# High mesothelin expression by immunohistochemistry predicts improved survival in pleural mesothelioma

**DOI:** 10.1111/his.14916

**Published:** 2023-04-11

**Authors:** Gerard J Chu, Anthony Linton, Steven Kao, Sonja Klebe, Stephen Adelstein, Dannel Yeo, John E J Rasko, Wendy A Cooper

**Affiliations:** ^1^ Gene and Stem Cell Therapy Program Centenary Institute University of Sydney Sydney NSW Australia; ^2^ Department of Clinical Immunology and Allergy Royal Prince Alfred Hospital Camperdown NSW Australia; ^3^ Faculty of Medicine and Health University of Sydney Sydney NSW Australia; ^4^ Department of Medical Oncology Concord Hospital Concord NSW Australia; ^5^ Asbestos Diseases Research Institute Concord NSW Australia; ^6^ Department of Medical Oncology Chris O'Brien Lifehouse Camperdown NSW Australia; ^7^ Department of Anatomical Pathology Flinders University and SA Pathology Adelaide SA Australia; ^8^ Cell and Molecular Therapies, Royal Prince Alfred Hospital Camperdown NSW Australia; ^9^ Department of Tissue Pathology and Diagnostic Oncology, NSW Health Pathology Royal Prince Alfred Hospital Camperdown NSW Australia; ^10^ School of Medicine Western Sydney University Sydney NSW Australia

**Keywords:** antibodies, chimeric antigen receptor T cells, epithelioid, expression, immunohistochemistry, mesothelin, mesothelioma, prognosis

## Abstract

**Aims:**

Mesothelin (MSLN) is a cancer‐associated antigen that is overexpressed in malignancies such as mesothelioma, pancreatic and ovarian cancer. It is also a target for novel personalised therapies, including antibodies, antibody–drug conjugates and chimeric antigen receptor T cells. Immunohistochemistry may predict those who would best respond to anti‐mesothelin therapies and guide decisions in therapeutic strategy. This study aimed to assess the intensity and distribution of MSLN immunostaining in mesothelioma, and to determine the prognostic value of MSLN expression by histochemical‐score (H‐score).

**Methods and results:**

The MN1 anti‐MSLN antibody was used to stain a formalin‐fixed paraffin‐embedded tissue microarray of histologically confirmed mesothelioma from 75 consecutive patients who had undergone pleurectomy with or without decortication. MSLN positivity, the staining intensity, distribution of staining and H‐score were evaluated. The correlation of H‐score with prognosis was investigated. Sixty‐six per cent of epithelioid tumours were MSLN‐positive (with expression in > 5% tumour cells). Of MSLN‐expressing epithelioid tumours, 70.4% had moderate (2+) or strong (3+) intensity MSLN immunostaining, although only 37% of samples had staining in ≥ 50% of tumour cells. In multivariate analysis, MSLN H‐score as a continuous variable and an H‐score ≥ 33 were independent predictors of improved survival (*P* = 0.04 and *P* < 0.001, respectively).

**Conclusions:**

MSLN expression was more heterogenous in epithelioid mesothelioma than reported previously. Therefore, it would be appropriate to perform an immunohistochemical assessment of MSLN expression to stratify and assess patient suitability for mesothelin‐targeted personalised therapies, such as chimeric antigen receptor T cells.

## Graphical abstract

MSLN immunostaining in an Australian cohort with epithelioid mesothelioma demonstrated inter‐patient heterogeneity of expression. Increased MSLN expression as measured by histochemical score was associated with increased survival. Immunohistochemistry (IHC) to MSLN should be performed to stratify and select eligible patients for personalised anti‐MSLN therapies.

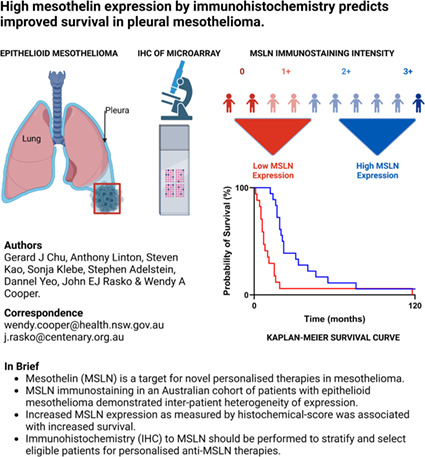

## Introduction

Mesothelin (MSLN) is a cancer‐associated antigen and a promising target for personalised therapies in diseases that overexpress MSLN, such as mesothelioma, pancreatic and ovarian cancer. MSLN is a membrane‐bound extracellular protein that functions as a carbohydrate‐binding lectin and binds to mucin 16 (MUC16), also known as cancer antigen 125.^1^ The MSLN precursor protein is cleaved to produce mature MSLN and another protein called megakaryocyte potentiating factor (MPF). MSLN is bound to the membrane by a glycophosphatidylinositol anchor but can be shed from the cell surface via several enzymes to release soluble MSLN‐related peptide (SMRP).^2^ Both MPF and SMRP are serum biomarkers of mesothelioma.^3^

The physiological function of MSLN is unknown; knockout (KO) mice have no discernible phenotype in health, development or reproduction.^4^ However, MSLN may have a role in the fibrotic response to injury, as MSLN KO mice have attenuated fibrosis in mouse models of fibrotic disease.^5^, ^6^, ^7^ In mouse models of human malignancy, MSLN's interaction with MUC16 has been associated with increased metastases.^8^ Mouse models have prompted the hypothesis that MSLN expression can lead to faster tumour growth, although this is not observed universally, and may depend upon the tumour location and the presence of a functioning immune system.^8^, ^9^, ^10^, ^11^

MSLN is a promising target for anti‐cancer personalised therapy with anticipated low toxicity due to its limited expression in healthy or essential tissues. MSLN is expressed in mesothelial cells of the pleura, pericardium and peritoneum, as well as in epithelial cells of the seminal vesicles, Hassal's corpuscles of the thymus, crypt cells of the tonsil, rectal mucosa, mucinous cells in the anal transitional epithelium and amnion/chorion of mature placenta.^12^, ^13^ MSLN can be expressed in tumours that arise from these tissues, such as mesothelioma. However, MSLN is also expressed in tumours that emerge from tissues that do not normally express MSLN, such as pancreatic cancer.

Immunohistochemistry (IHC) to MSLN is occasionally employed to distinguish epithelioid mesothelioma from other diagnoses.^14^ In addition, recent evidence indicates that MSLN expression by IHC may be valuable in selecting patients for anti‐MSLN targeted therapies. Anetumab ravtansine, an anti‐mesothelin antibody conjugated to a maytansine derivative tubulin inhibitor, was recently investigated in a randomised, open‐label Phase 2 trial in mesothelioma.^15^ While anetumab ravtansine was not superior to vinorelbine, a *post‐hoc* analysis revealed that progression‐free and overall survival was substantially higher in patients treated with anetumab ravtansine who had MSLN membrane staining in > 50% of tumour cells compared to those who had MSLN staining in only 30–50% of tumour cells.^15^ This trial was the first, to our knowledge, to highlight the value of IHC in patient selection for an anti‐MSLN targeted therapy.

This prompted a review of the role of IHC in other Phase I/II trials of anti‐MSLN‐targeted therapies; it is conspicuous that many trials have incomplete IHC data on MSLN expression. This is the case for the anti‐MSLN SS1 antibody conjugated to the pseudomonas exotoxin (SS1P),^16^ the humanised anti‐MSLN antibody amatuximab,^17^ and another antibody–drug conjugate targeting MSLN, DMOT4039A.^18^ MSLN IHC is also not currently considered essential in Phase I/II trials of anti‐MSLN cellular therapies. Only one trial of a genetically modified T cell receptor fusion construct required 2+/3+ MSLN expression in ≥ 50% viable tumour cells by IHC as its sole inclusion criterion relating to MSLN expression.^19^ SMRP is often used as an alternative inclusion criterion,^20^ while other anti‐MSLN CAR T cell trials have not required MSLN expression for patient inclusion.^21^, ^22^ The lack of IHC in assessing eligibility for anti‐MSLN CAR T cell trials may be due to the presumption that MSLN‐expression is universally present and at high intensity, as reported in the earliest literature of MSLN immunostaining in mesothelioma.^23^, ^24^, ^25^, ^26^

However, the lack of standardisation in the measurement, scoring and interpretation of MSLN immunostaining has led to variability in the literature relating to MSLN expression by IHC. Different patient cohorts may also contribute to the variability, as MSLN is more likely to be expressed in epithelioid than sarcomatoid mesotheliomas.^12^, ^27^ However, even when focusing upon the epithelioid subtype, reported rates of MSLN expression among tumour specimens range from 64%^13^ to 100%.^23^ When assessing the distribution of MSLN within a single tumour specimen, between 53.6%^28^ and 83.9%^29^ of epithelioid mesothelioma specimens have diffuse expression in ≥ 50% of the tumour cells. Finally, when assessing the intensity of MSLN immunostaining, the values of moderate (2+) or strong (3+) intensity immunostaining by IHC vary between 52%^13^ and 70%^27^ in epithelioid mesothelioma.

MSLN expression may also have prognostic significance. One group showed an improved overall survival in tumours diffusely positive for MSLN staining compared to MSLN‐negative/partial staining tumours.^12^ However, this study also included sarcomatoid tumours that are known to have both lower levels of MSLN expression and a poorer prognosis.^27^ Only one study has analysed MSLN expression by IHC and controlled for other confounders including tumour subtype in a multivariate analysis.^27^ This study identified that high expression of MSLN led to a reduced hazard ratio (HR) of death of 0.53 [95% confidence interval (CI) = 0.31–0.91].^27^ In contrast to these data, a large single‐centre analysis of stage III epithelioid tumours found that T3 tumours had a greater percentage of strong MSLN immunostaining compared to T2 tumours (51 versus 25%), suggesting that invasiveness was associated with increased MSLN expression.^11^

Due to the uncertainty regarding the expression and prognostic significance of MSLN in mesothelioma, we aimed to assess these factors in a cohort of patients with mesothelioma to determine which patients are suitable for anti‐MSLN targeted therapies and to assist in the design of future clinical trials.

## Materials and methods

### Patient cohort

Tumour samples and clinical follow‐up data were collected from 75 consecutive patients diagnosed with pleural mesothelioma who had undergone pleurectomy with or without decortication at Royal Prince Alfred Hospital, Sydney, between 1992 and 2007, and published previously.^30^, ^31^ The pathological diagnosis of mesothelioma and the mesothelioma subtype was established by a pathologist (S.K.) using the World Health Organisation classification.^32^

### Tumour samples

Tissue microarrays were constructed using four 1 mm diameter donor cores of tumour. At least three cores were also taken from normal mesothelium, tonsil, breast, kidney, liver, lung, lymph node and prostate specimens as controls. The cores were re‐embedded in paraffin blocks and sectioned at 4 μm thickness and mounted on glass slides. The analysis of these samples was approved by the University of Sydney Human Ethics Research Committee (Project 2018/730).

### Immunohistochemistry

Tissue sections were placed in a 60°C oven to melt paraffin. The slides were then deparaffinised in xylene and then rehydrated through decreasing concentrations of alcohol. Antigen retrieval was performed in a pressurised decloaking chamber (Biocare Medical, Pacheco, CA, USA) in pH 9 antigen retrieval solution (Dako, Santa Clara, CA, USA) at 95°C for 30 min. Specimens were stained using a Dako Autostainer. Endogenous peroxidase was inactivated with 3% hydrogen peroxide for 5 min. Tissues were blocked with serum‐free protein block (Dako) followed by air‐drying. Tissues were stained for 30 min at room temperature with MN1 anti‐mesothelin antibody (Rockland Immunochemicals, Pottstown, PA, USA) at a concentration of 1 μg/ml, diluted in Dako antibody diluent. Envision mouse horseradish peroxidase (HRP)‐labelled polymer secondary (Dako) was added for 30 min. Finally, 3,3′‐diaminobenzidine (DAB) substrate was added for 10 min. Slides were counterstained with Harris haematoxylin. Images were acquired with an Olympus DP73 model (Olympus, Shinjuku City, Japan) at a 35 mm focal length and 1600 × 1200 pixel resolution at 200× and 400× magnification.

### Scoring

MSLN staining was independently assessed by two pathologists (G.J.C. and W.A.C.) and the average score calculated for each patient without knowledge of patient clinical outcomes. The predominant staining intensity was scored between 0 and 3, where 0 is no staining, 1 is low‐intensity staining, 2 is medium‐intensity staining and 3 is high‐intensity staining. The percentage of tumour that stained positively was calculated and the product of the staining intensity and percentage of positive tumour calculated to give a H‐score out of 300, as described previously.^27^

### Statistical analysis

Statistical analysis was performed using GraphPad Prism version 9.4.1 (GraphPad Software, San Diego, CA, USA). The Mann–Whitney test was used to examine correlations between survival and available clinical characteristics, including age, sex and histological subtype. Comparisons of H‐score across histological subtypes were made by the Mann–Whitney test. When assessing the presence or absence of MSLN immunostaining, differences between histological subtypes were calculated by Fisher's exact test (two‐sided). Differences in survival on Kaplan–Meier curves were calculated by the log‐rank (Mantel–Cox) test. Multivariate analyses and adjustment for age, sex and histology were performed by Cox proportional hazards regression.

## Results

### Patient samples

Of the 75 patient specimens, eight could not be analysed due to technical issues: five had inadequate tissue for analysis due to tissue loss or folding, one sample had no detectable malignancy in the four sections, and two had a mixture of limited tissue or limited mesothelioma for analysis.

### Patient clinical characteristics

The clinical characteristics of the 67 patients are summarised in Table 1. A significant difference in median overall survival between the epithelioid and non‐epithelioid tumours was observed, consistent with the known poor prognosis associated with sarcomatoid tumours. Few patients were eligible for current standard‐of‐care chemotherapy because samples predated the advent of cisplatin/pemetrexed standard‐of‐care in Australia.^33^ Surgery in this cohort was palliative pleurectomy with or without decortication, and pre‐dates extended pleurectomy and decortication procedures currently performed. As such, no neoadjuvant/adjuvant therapy, including chemotherapy or radiotherapy, was included as part of the treatment regimen. A minority of patients may have received limited palliative chemotherapy or radiotherapy to treat their symptoms, but this was not part of multi‐modal therapy and in insufficient numbers to perform a subgroup analysis or determine whether these factors should be included in the Cox multivariate model. The cohort is therefore highly homogenous, albeit with a lower overall survival compared to recent publications.

**Table 1:** Clinicopathological features and median overall survival of patients included in the tumour microarray analyses

Variable	*n* (%)	Median OS in months (95% CI)	*P*‐value
Overall group	67 (100)	9 (6–12)	
Age (years)			
< 65	31 (46)	9 (6–19)	0.168
≥ 65	36 (54)	8.5 (5–13)	
Sex			
Male	53 (79)	9 (6–13)	0.2579
Female	14 (21)	8.5 (5–55)	
Histological subtype			
Epithelioid	35 (52)	16 (11–20)	<0.0001*
Non‐epithelioid	32 (48)	5.5 (4–8)	
Biphasic	22 (33)	6 (3–9)	
Sarcomatoid	10 (15)	4 (1–8)	

* Comparing epithelioid and non‐epithelioid subtypes.

OS, overall survival; CI, confidence interval.

### MSLN immunostaining in normal tissues

Membranous MSLN immunostaining was observed in normal mesothelium, with staining intensity varying from intense to trace staining. Weak to moderate membranous MSLN staining was also detected in the tonsillar crypt cells but not in tonsillar lymphoid tissue. MSLN immunostaining was not detected in breast, kidney, liver, lung, lymph node or prostate.

### MSLN immunostaining in mesothelioma

**Figure 1:**
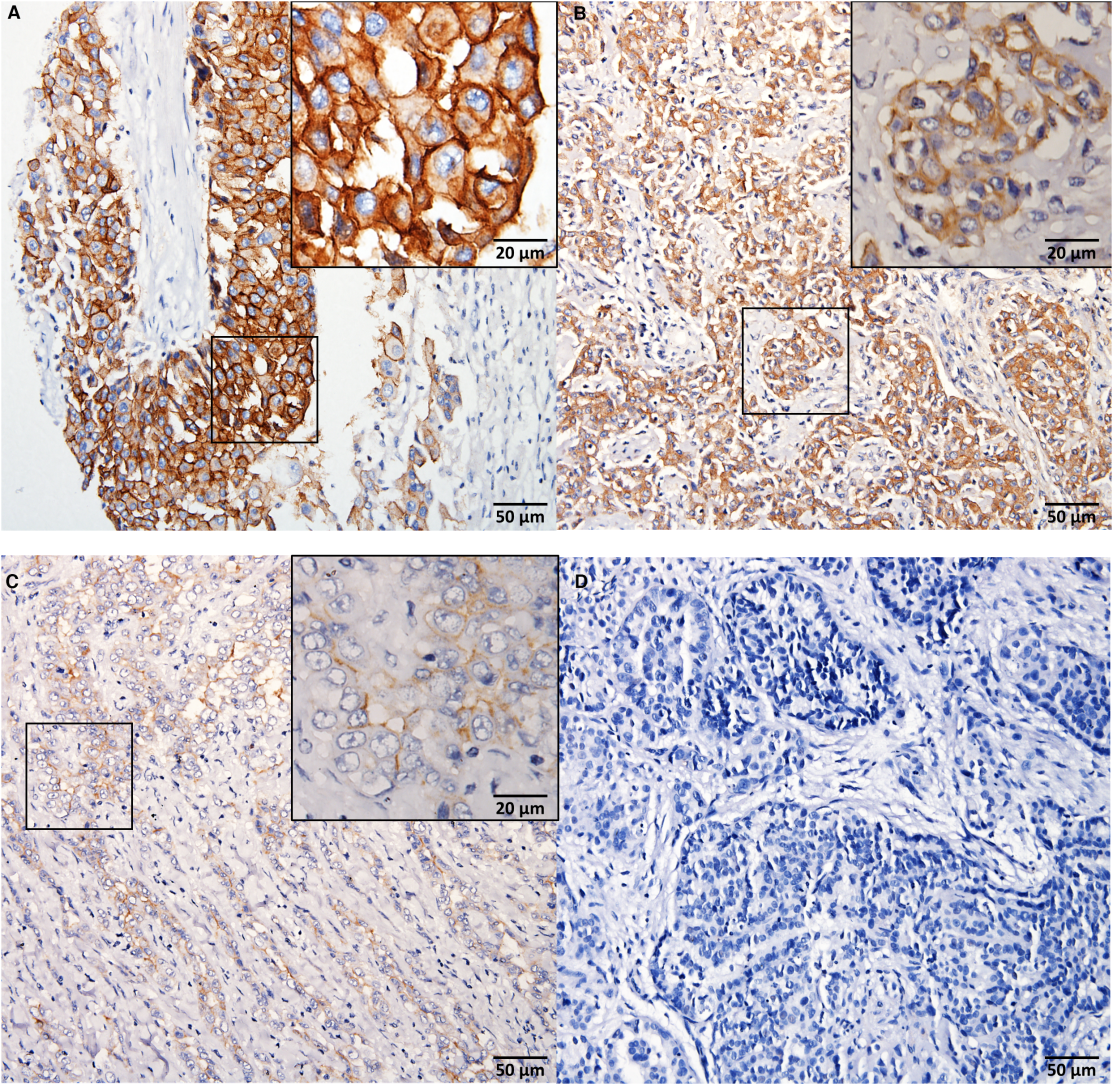
There is a spectrum of mesothelin (MSLN) immunostaining intensity in epithelioid mesothelioma. **A,** High‐intensity MSLN immunostaining (membranous and cytoplasmic immunoreactivity) in epithelioid mesothelioma. **B,** Moderate intensity MSLN immunostaining (membranous and cytoplasmic immunoreactivity) in epithelioid mesothelioma. **C,** Weak intensity MSLN immunostaining (membranous immunoreactivity) in epithelioid mesothelioma. **D,** Epithelioid mesothelioma lacking MSLN expression. [Color figure can be viewed at wileyonlinelibrary.com]

Membranous staining with fainter cytoplasmic staining was the most common MSLN immunostaining pattern in epithelioid mesothelioma (Figure 1A,B). Overall, 39 of 67 (58%) of mesothelioma tissues stained positive for MSLN at any level of expression. Thirty‐two of 67 (48%) mesothelioma patient samples stained positive for MSLN using a threshold for positivity of immunostaining in ≥ 5% of tumour cells, a threshold reported previously.^12^ A substantially greater proportion of the epithelioid subtype stained positive (23 of 35, 66%) compared to the non‐epithelioid subtypes (nine of 32, 28%) (*P* = 0.003). In the non‐epithelioid subtypes, only the biphasic specimens with epithelioid features stained positive for mesothelin (nine of 22, 41%), and all sarcomatoid tissues (none of 10) were negative for MSLN expression. The intensity and distribution of MSLN immunostaining in positive mesothelioma specimens are summarised in Table 2. The median H‐score for epithelioid tumours was 33. This was significantly higher than non‐epithelioid tumours (median H‐score = 0, *P* < 0.001) and the difference was significant for both sarcomatoid tumours (median H‐score = 0, *P* < 0.001) and biphasic tumours (median H‐score = 1.5, *P* = 0.002). There was no significant difference in specimen age between epithelioid tumours of differing MSLN staining intensity (data not shown).

**Table 2:** The intensity and distribution of MSLN immunostaining in epithelioid and biphasic mesotheliomas that express MSLN

	Epithelioid (%) *n* = 27	Biphasic (%) *n* = 12
Intensity		
Strong	3 (11.1)	0
Moderate	16 (59.3)	5 (41.7)
Weak	8 (29.6)	7 (58.3)
Distribution		
≥ 50%	10 (37.0)	0
< 50%	17 (63.0)	12 (100.0)

MSLN, mesothelin.

### MSLN immunostaining and prognosis

**Figure 2:**
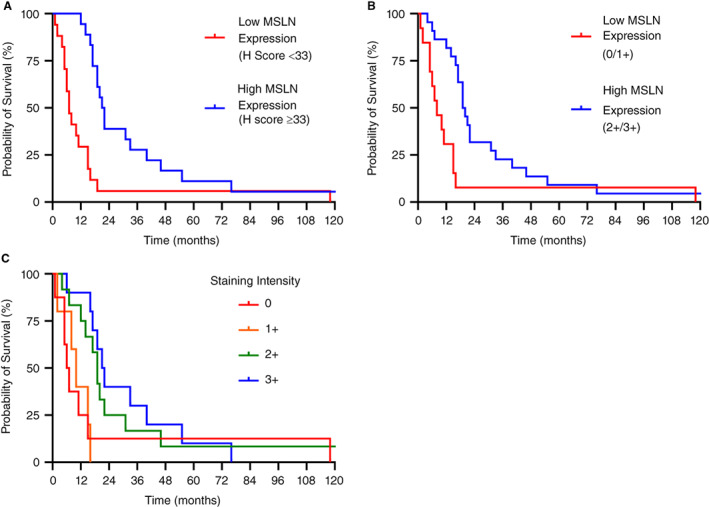
High mesothelin (MSLN) expression is associated with an increased median survival in epithelioid mesothelioma. **A,** Kaplan–Meier survival curve of patients with epithelioid mesothelioma and high MSLN expression (H score ≥ 33) and low MSLN expression (H score < 33). **B,** Kaplan–Meier survival curve of patients with epithelioid mesothelioma and high MSLN expression (staining intensity 2+/3+) and low MSLN expression (staining intensity 0/1+). **C,** Kaplan–Meier survival curve of patients with epithelioid mesothelioma with varying staining intensity (0, 1+, 2+, 3+). [Color figure can be viewed at wileyonlinelibrary.com]

Given the difference in prognosis between epithelioid and non‐epithelioid subtypes, a survival analysis was performed on each subtype separately. Epithelioid tumours were divided by their median H‐score of 33 into a MSLN‐high group (H‐score ≥ 33) and MSLN‐low group (H‐score < 33), and their survival compared (Figure 2A). The MSLN‐high group had a significantly higher median survival of 21.5 months (95% CI = 17–40 months) compared to 7 months in the MSLN‐low group (95% CI = 5–15 months) (log‐rank *P* < 0.001) (Figure 2A). The median H score for non‐epithelioid tumours was 0, and used to divide the non‐epithelioid group into a group with no MSLN expression (H‐score = 0) and MSLN‐expressing group (H‐score ≥ 1), and no difference in survival was noted between these groups. However, the one patient with biphasic mesothelioma and an H‐score ≥ 33 had an extended survival of 19 months. Similar results were seen when evaluating the specimen's maximum staining intensity. The median survival was 20.5 months (95% CI = 17–33 months) for epithelioid tumours with 2+/3+ staining intensity compared to 8 months (95% CI = 5–15 months) for tumours with 0 or 1+ staining intensity (log‐rank, *P =* 0.007) (Figure 2B). Patient survival also increased with each increment in MSLN staining intensity (log‐rank test for trend, *P* = 0.023) (Figure 2C).

A Cox multivariate analysis was performed on the entire cohort, adjusting for age, sex and tumour histology. MSLN positivity (expression in ≥ 5% tumour) was associated with a reduced hazard ratio, but this did not reach statistical significance (*P* = 0.114, HR = 0.587, 95% CI = 0.3025–1.139). However, both H‐score as a continuous variable and an H‐score ≥ 33 were independent prognostic markers of longer survival (*P* = 0.040, HR = 0.994, 95% CI = 0.989–0.999 and *P* < 0.001, HR = 0.25 95% CI = 0.117–0.516, respectively). Of note, the HR for a continuous variable is calculated for each integer difference across the entire range of H‐scores between 0 and 300. As such, a difference between an H‐score of 1 and 2 would not have any clinical significance, whereas larger differences in H‐score, such as between 1 and 100, would be clinically meaningful. Similarly, a staining intensity of 2+ or 3+ was also associated with a reduced HR (*P* = 0.003, HR = 0.384, 95% CI = 0.202–0.719).

## Discussion

Although MSLN is overexpressed in mesothelioma, our cohort demonstrates inter‐patient heterogeneity in MSLN expression. Sixty‐six per cent of our epithelioid cohort were MSLN‐positive. Of the MSLN‐staining tissues, the majority (70.4%) were moderate or strong in intensity, but only a minority (37%) had expression in ≥ 50% of tumour cells. These data are consistent with the degree of MSLN‐positivity seen in a comprehensive atlas of MSLN immunostaining.^13^ However, the rates of positivity are substantially lower than reported in earlier papers using a different 5B2 anti‐MSLN antibody.^23^, ^24^ A difference in monoclonal antibody sensitivity and concentration could explain the discrepancy. However, the only head‐to‐head comparisons between 5B2 and MN1 show equivalent or superior performance for MN1 in immunohistochemistry^12^ and other *in‐vitro* techniques^34^ compared to 5B2. The MN1 antibody was chosen for this study as it binds to the N‐terminal region of mature MSLN,^34^ a region that is targeted by antibodies (such as amatuximab), antibody–drug conjugates (such as the SS1P) and SS1 CAR T cells, and is therefore a relevant antibody for these targeted therapies. In contrast, it is not known to which epitope 5B2 binds.^34^ There are two clear implications of these data. First, MSLN‐staining would be advised to select patients for anti‐MSLN targeted therapies in mesothelioma. Secondly, there is a risk of antigen‐escape, which is the selective survival of antigen‐negative tumour, if only a minority of cases have MSLN expression in ≥ 50% of tumour cells.

Our data also confirmed that higher MSLN expression is associated with improved patient survival in epithelioid mesothelioma. The one patient with biphasic mesothelioma who had a high H‐score also had increased survival, but more cases are required to determine whether MSLN expression also has prognostic relevance to non‐epithelioid subtypes. While MSLN positivity (≥ 5% of tumour) was not an independent prognostic marker, the degree of MSLN expression, measured by H‐score as a continuous variable or an elevated H‐score ≥ 33, and a 2+/3+ staining intensity, were independent prognostic markers of improved survival. While these results are consistent with the prognostic data of MSLN expression in mesothelioma presented elsewhere,^12^, ^27^ we recommend that they be validated in a prospective trial and the reliability of H‐score testing assessed to confirm the findings of this study. Although one study proposed that higher MSLN expression is associated with greater invasiveness in mesothelioma, as assessed by tumour T‐stage, that study did not include survival data.^11^ As staging is clinically difficult to perform in the absence of radical surgery there were no comprehensive disease‐staging data available for our cohort, and analysis of survival and the degree of MSLN expression at differing disease stages was precluded. While higher serum SMRP is related to a poorer clinical outcome,^35^ SMRP levels may reflect tumour size or the extent of enzymatic shedding rather than the degree of surface MSLN expression. Therefore, this could still be consistent with our data. As MSLN is expressed on healthy pleural tissues, an inverse relationship between MSLN immunoreactivity and cellular atypia has been proposed,^36^ and it is possible that MSLN expression could be reduced as tumour cells become less differentiated, leading to an association with poorer prognosis. Another mesothelial marker, podoplanin (D2‐40), is commonly co‐expressed with MSLN in mesothelioma, has an association with improved prognosis and may have an inverse relationship with atypia.^36^ In addition, the mesothelial marker, Wilms’ tumour 1 (WT1), is commonly coexpressed with MSLN and immunoreactivity is similarly associated with improved survival in mesothelioma.^29^, ^37^ Conversely, the mesothelial marker, calretinin, can be coexpressed with MSLN in epithelioid mesothelioma, but its association with prognosis and atypia is unclear.^29^, ^36^ A correlation between MSLN and these other mesothelial markers in our cohort is the subject of further work. Loss of MSLN mRNA expression was also identified in a poor prognostic subgroup of mesothelioma in an integrative, multiplatform genome analysis of The Cancer Genome Atlas specimens.^38^ Other associated characteristics of this MSLN‐low group include greater epithelial–mesenchymal transition, and while there was no relationship between MSLN and *BAP1* mutations there was a greater number of *LATS2* mutations and *CDKN2A* homozygous deletions in this MSLN‐low group.^38^ Each of these associations could explain the prognostic significance of MSLN expression in mesothelioma.^38^, ^39^, ^40^ Of interest, while an association of MSLN with improved prognosis is noted in mesothelioma, this relationship is not seen in all MSLN‐expressing diseases.^13^, ^41^ The unfortunate implication is that mesothelioma patients with a risk of poor prognosis are less likely to be suitable for anti‐MSLN targeted therapies. In addition, trials that select patients with high MSLN‐expression will be biased towards patients who have better survival, and circumspection is required if survival is compared to historical values, rather than compared to control patients with similar degrees of MSLN expression.

To conclude, in this Australian cohort the percentage of MSLN‐expressing mesothelioma tissues and the distribution of MSLN were lower than reported elsewhere. Of clinical significance, this predicts that heterogenous expression and antigen‐escape could be obstacles to anti‐MSLN therapy. Therefore, it is advised that IHC be used as a diagnostic assay to stratify and select eligible patients with mesothelioma for personalised anti‐MSLN therapies in future clinical trials.

## Conflicts of interest

G.C. is a recipient of the PhD completion Scholarship in Immunotherapies for Treating Mesothelin‐Positive Cancers (2021–22) funded by a grant awarded to J.R. from CSR Limited.

## Data Availability

The data that support the findings of this study are available on request from the corresponding author. The data are not publicly available due to privacy or ethical restrictions.

## References

[1] Kaneko O , Gong L , Zhang J *et al*. A binding domain on mesothelin for CA125/MUC16. J. Biol. Chem. 2009; 284; 3739–3749. 19075018 10.1074/jbc.M806776200 PMC2635045

[2] Liu X , Chan A , Tai CH , Andresson T , Pastan I . Multiple proteases are involved in mesothelin shedding by cancer cells. Commun. Biol. 2020; 3; 728. 33262421 10.1038/s42003-020-01464-5 PMC7708464

[3] Hollevoet K , Nackaerts K , Thimpont J *et al*. Diagnostic performance of soluble mesothelin and megakaryocyte potentiating factor in mesothelioma. Am. J. Respir. Crit. Care Med. 2010; 181; 620–625. 20075387 10.1164/rccm.200907-1020OC

[4] Bera TK , Pastan I . Mesothelin is not required for normal mouse development or reproduction. Mol. Cell. Biol. 2000; 20; 2902–2906. 10733593 10.1128/mcb.20.8.2902-2906.2000 PMC85523

[5] Rinkevich Y , Mori T , Sahoo D , Xu PX , Bermingham JR Jr , Weissman IL . Identification and prospective isolation of a mesothelial precursor lineage giving rise to smooth muscle cells and fibroblasts for mammalian internal organs, and their vasculature. Nat. Cell Biol. 2012; 14; 1251–1260. 23143399 10.1038/ncb2610 PMC3685475

[6] Iwaisako K , Jiang C , Zhang M *et al*. Origin of myofibroblasts in the fibrotic liver in mice. Proc. Natl. Acad. Sci. U.S.A. 2014; 111; E3297–E3305. 25074909 10.1073/pnas.1400062111 PMC4136601

[7] Koyama Y , Wang P , Liang S *et al*. Mesothelin/mucin 16 signaling in activated portal fibroblasts regulates cholestatic liver fibrosis. J. Clin. Invest. 2017; 127; 1254–1270. 28287406 10.1172/JCI88845 PMC5373891

[8] Avula LR , Rudloff M , El‐Behaedi S *et al*. Mesothelin enhances tumor vascularity in newly forming pancreatic peritoneal metastases. Mol. Cancer Res. 2020; 18; 229–239. 31676721 10.1158/1541-7786.MCR-19-0688 PMC8139242

[9] Zervos E , Agle S , Freistaedter AG , Jones GJ , Roper RL . Murine mesothelin: characterization, expression, and inhibition of tumor growth in a murine model of pancreatic cancer. J. Exp. Clin. Cancer Res. 2016; 35; 39. 26931187 10.1186/s13046-016-0314-2 PMC4774190

[10] Bharadwaj U , Marin‐Muller C , Li M , Chen C , Yao Q . Mesothelin confers pancreatic cancer cell resistance to TNF‐alpha‐induced apoptosis through Akt/PI3K/NF‐kappaB activation and IL‐6/Mcl‐1 overexpression. Mol. Cancer 2011; 10; 106. 21880146 10.1186/1476-4598-10-106 PMC3175472

[11] Servais EL , Colovos C , Rodriguez L *et al*. Mesothelin overexpression promotes mesothelioma cell invasion and MMP‐9 secretion in an orthotopic mouse model and in epithelioid pleural mesothelioma patients. Clin. Cancer Res. 2012; 18; 2478–2489. 22371455 10.1158/1078-0432.CCR-11-2614 PMC3759995

[12] Inaguma S , Wang Z , Lasota J *et al*. Comprehensive immunohistochemical study of mesothelin (MSLN) using different monoclonal antibodies 5B2 and MN‐1 in 1562 tumors with evaluation of its prognostic value in malignant pleural mesothelioma. Oncotarget 2017; 8; 26744–26754. 28460459 10.18632/oncotarget.15814 PMC5432294

[13] Weidemann S , Gagelmann P , Gorbokon N *et al*. Mesothelin expression in human tumors: a tissue microarray study on 12,679 tumors. Biomedicine 2021; 9; 9. 10.3390/biomedicines9040397 PMC8067734 33917081

[14] Husain AN , Colby TV , Ordonez NG *et al*. Guidelines for pathologic diagnosis of malignant mesothelioma 2017 update of the consensus statement from the International Mesothelioma Interest Group. Arch. Pathol. Lab. Med. 2018; 142; 89–108. 28686500 10.5858/arpa.2017-0124-RA

[15] Kindler HL , Novello S , Bearz A *et al*. Anetumab ravtansine versus vinorelbine in patients with relapsed, mesothelin‐positive malignant pleural mesothelioma (ARCS‐M): a randomised, open‐label phase 2 trial. Lancet Oncol. 2022; 23; 540–552. 35358455 10.1016/S1470-2045(22)00061-4 PMC10512125

[16] Hassan R , Sharon E , Thomas A *et al*. Phase 1 study of the antimesothelin immunotoxin SS1P in combination with pemetrexed and cisplatin for front‐line therapy of pleural mesothelioma and correlation of tumor response with serum mesothelin, megakaryocyte potentiating factor, and cancer antigen 125. Cancer 2014; 120; 3311–3319. 24989332 10.1002/cncr.28875 PMC6334650

[17] Hassan R , Kindler HL , Jahan T *et al*. Phase II clinical trial of amatuximab, a chimeric antimesothelin antibody with pemetrexed and cisplatin in advanced unresectable pleural mesothelioma. Clin. Cancer Res. 2014; 20; 5927–5936. 25231400 10.1158/1078-0432.CCR-14-0804 PMC4252585

[18] Weekes CD , Lamberts LE , Borad MJ *et al*. Phase I study of DMOT4039A, an antibody‐drug conjugate targeting Mesothelin, in patients with unresectable pancreatic or platinum‐resistant ovarian cancer. Mol. Cancer Ther. 2016; 15; 439–447. 26823490 10.1158/1535-7163.MCT-15-0693

[19] Hong DJ , M , Tanyi JL *et al*. Abstract CT105: Preliminary safety and efficacy of gavocabtagene autoleucel (gavo‐cel, TC‐210), a T cell receptor fusion construct (TRuC™), in patients with treatment refractory mesothelin overexpressing solid tumors. Proceedings of the American Association for Cancer Research Annual Meeting 2021; 2021 Apr 10–15 and May 17–21 Philadelphia (PA); 2021. Cancer Res. 2021; 81(13_Supplement); CT105.

[20] Adusumilli PS , Zauderer MG , Riviere I *et al*. A phase I trial of regional mesothelin‐targeted CAR T‐cell therapy in patients with malignant pleural disease, in combination with the anti‐PD‐1 agent pembrolizumab. Cancer Discov. 2021; 11; 2748–2763. 34266984 10.1158/2159-8290.CD-21-0407 PMC8563385

[21] Phase I study of autologous T cells bearing fully‐humanized chimeric antigen receptor targeting mesothelin in mesothelin expressing cancers. 2021 https://www.med.upenn.edu/cellicon2021/assets/user‐content/documents/tanyi.pdf. 10.1016/j.ymthe.2026.01.021 PMC13154321 41566776

[22] Beatty GL , O'Hara MH , Lacey SF *et al*. Activity of mesothelin‐specific chimeric antigen receptor T cells against pancreatic carcinoma metastases in a phase 1 trial. Gastroenterology 2018; 155; 29–32. 29567081 10.1053/j.gastro.2018.03.029 PMC6035088

[23] Ordonez NG . The immunohistochemical diagnosis of mesothelioma: a comparative study of epithelioid mesothelioma and lung adenocarcinoma. Am. J. Surg. Pathol. 2003; 27; 1031–1051. 12883236 10.1097/00000478-200308000-00001

[24] Ordonez NG . The diagnostic utility of immunohistochemistry in distinguishing between mesothelioma and renal cell carcinoma: a comparative study. Hum. Pathol. 2004; 35; 697–710. 15188136 10.1016/j.humpath.2003.11.013

[25] Galloway ML , Murray D , Moffat DF . The use of the monoclonal antibody mesothelin in the diagnosis of malignant mesothelioma in pleural biopsies. Histopathology 2006; 48; 767–769. 10.1111/j.1365-2559.2005.02279.x 16681698

[26] Miettinen M , Sarlomo‐Rikala M . Expression of calretinin, thrombomodulin, keratin 5, and mesothelin in lung carcinomas of different types: an immunohistochemical analysis of 596 tumors in comparison with epithelioid mesotheliomas of the pleura. Am. J. Surg. Pathol. 2003; 27; 150–158. 12548160 10.1097/00000478-200302000-00002

[27] Vizcaya D , Farahmand B , Walter AO *et al*. Prognosis of patients with malignant mesothelioma by expression of programmed cell death 1 ligand 1 and mesothelin in a contemporary cohort in Finland. Cancer Treat Res Commun 2020; 25; 100260. 33310366 10.1016/j.ctarc.2020.100260

[28] Kushitani K , Takeshima Y , Amatya VJ , Furonaka O , Sakatani A , Inai K . Immunohistochemical marker panels for distinguishing between epithelioid mesothelioma and lung adenocarcinoma. Pathol. Int. 2007; 57; 190–199. 17316414 10.1111/j.1440-1827.2007.02080.x

[29] Eguchi T , Kadota K , Mayor M *et al*. Cancer antigen profiling for malignant pleural mesothelioma immunotherapy: expression and coexpression of mesothelin, cancer antigen 125, and Wilms tumor 1. Oncotarget 2017; 8; 77872–77882. 29100432 10.18632/oncotarget.20845 PMC5652821

[30] Kao SC , Cheng YY , Williams M *et al*. Tumor suppressor microRNAs contribute to the regulation of PD‐L1 expression in malignant pleural mesothelioma. J. Thorac. Oncol. 2017; 12; 1421–1433. 28629895 10.1016/j.jtho.2017.05.024

[31] Linton A , Cheng YY , Griggs K *et al*. An RNAi‐based screen reveals PLK1, CDK1 and NDC80 as potential therapeutic targets in malignant pleural mesothelioma. Br. J. Cancer 2014; 110; 510–519. 24327015 10.1038/bjc.2013.731 PMC3899767

[32] Travis WBE , Muller‐Hermelink HK , Harris CC eds. World Health Organisation classification of tumours. Pathology and genetics of tumours of the lung, pleura, thymus and heart. Lyon: IARC Press, 2004.

[33] Vogelzang NJ , Rusthoven JJ , Symanowski J *et al*. Phase III study of pemetrexed in combination with cisplatin versus cisplatin alone in patients with malignant pleural mesothelioma. J. Clin. Oncol. 2003; 21; 2636–2644. 12860938 10.1200/JCO.2003.11.136

[34] Onda M , Willingham M , Nagata S *et al*. New monoclonal antibodies to mesothelin useful for immunohistochemistry, fluorescence‐activated cell sorting, Western blotting, and ELISA. Clin. Cancer Res. 2005; 11; 5840–5846. 16115924 10.1158/1078-0432.CCR-05-0578

[35] Tian L , Zeng R , Wang X *et al*. Prognostic significance of soluble mesothelin in malignant pleural mesothelioma: a meta‐analysis. Oncotarget 2017; 8; 46425–46435. 28507279 10.18632/oncotarget.17436 PMC5542278

[36] Sandeck HP , Røe OD , Kjærheim K , Willén H , Larsson E . Re‐evaluation of histological diagnoses of malignant mesothelioma by immunohistochemistry. Diagn. Pathol. 2010; 5; 47. 20602796 10.1186/1746-1596-5-47 PMC2915960

[37] Cedrés S , Montero MA , Zamora E *et al*. Expression of Wilms’ tumor gene (WT1) is associated with survival in malignant pleural mesothelioma. Clin. Transl. Oncol. 2014; 16; 776–782. 24323466 10.1007/s12094-013-1146-6

[38] Hmeljak J , Sanchez‐Vega F , Hoadley KA *et al*. Integrative molecular characterization of malignant pleural mesothelioma. Cancer Discovery 2018; 8; 1548–1565. 30322867 10.1158/2159-8290.CD-18-0804 PMC6310008

[39] Singhi AD , Krasinskas AM , Choudry HA *et al*. The prognostic significance of BAP1, NF2, and CDKN2A in malignant peritoneal mesothelioma. Mod. Pathol. 2016; 29; 14–24. 26493618 10.1038/modpathol.2015.121

[40] Tranchant R , Quetel L , Tallet A *et al*. Co‐occurring mutations of tumor suppressor genes, LATS2 and NF2, in malignant pleural mesothelioma. Clin. Cancer Res. 2017; 23; 3191–3202. 28003305 10.1158/1078-0432.CCR-16-1971

[41] Nahm CB , Turchini J , Jamieson N *et al*. Biomarker panel predicts survival after resection in pancreatic ductal adenocarcinoma: A multi‐institutional cohort study. Eur. J. Surg. Oncol. 2019; 45; 218–224. 30348604 10.1016/j.ejso.2018.10.050

